# Isometric skeletal muscle contractile properties in common strains of male laboratory mice

**DOI:** 10.3389/fphys.2022.937132

**Published:** 2022-10-04

**Authors:** Everett C. Minchew, Nicholas C. Williamson, Andrew T. Readyoff, Joseph M. McClung, Espen E. Spangenburg

**Affiliations:** ^1^ Department of Physiology, East Carolina University Brody School of Medicine, Greenville, NC, United States; ^2^ East Carolina University, East Carolina Diabetes and Obesity Institute, Greenville, NC, United States; ^3^ East Carolina Heart Institute, Greenville, NC, United States

**Keywords:** skeletal muscle, force, mouse model, muscle mass, genetics

## Abstract

Assessing contractile function of skeletal muscle in murine models is a commonly employed laboratory technique that investigators utilize to measure the impact of genetic manipulations, drug efficacy, or other therapeutic interventions. Often overlooked is the potential for the strain of the mouse to influence the functional properties of the skeletal muscle. Thus, we sought to characterize commonly assessed isometric force measures in the hindlimb muscles across a variety of mouse strains. Using 6-8-week-old male mice, we measured isometric force, fatigue susceptibility, relaxation kinetics, muscle mass, myofiber cross-sectional area, and fiber type composition of the extensor digitorum longus (EDL) and soleus muscles in C57BL/6NJ, BALB/cJ, FVB/NJ, C57BL/6J, and C57BL/10 mice. The data demonstrate both unique differences and a number of similarities between both muscles in the various genetic backgrounds of mice. Soleus muscle specific force (i.e., force per unit size) exhibited higher variation across strains while specific force of the EDL muscle exhibited minimal variation. In contrast, absolute force differed only in a few mouse strains whereas analysis of muscle morphology revealed many distinctions when compared across all the groups. Collectively, the data suggest that the strain of the mouse can potentially influence the measured biological outcome and may possibly promote a synergistic effect with any genetic manipulation or therapeutic intervention. Thus, it is critical for the investigator to carefully consider the genetic background of the mouse used in the experimental design and precisely document the strain of mouse employed during publication.

## Introduction

A primary role of skeletal muscle is the generation of force and ultimately movement. Force production is achieved via the interaction between the central nervous system and excitation contraction-coupling (ECC) mechanisms intrinsic to the muscle cell. Assessment of force production is the gold standard for measuring physiological function of the skeletal muscle. Skeletal muscle function is a critical predictor of mortality in humans (Li et al., 2018; Wang et al., 2019; Knowles et al., 2021), thus pre-clinical animal models are often employed to examine contractile properties such as isometric force production, fatigue resistance, and contraction kinetics (Brooks and Faulkner, 1988; Westerblad et al., 2010). Contractile force is induced by activation of ECC and is regulated by various factors including muscle size and fiber type composition (Fitts, 1994). Collectively, there are several anatomical and/or intracellular mechanisms that can affect contractile function of skeletal muscle.

Investigations have extensively leveraged the mouse (*Mus musculus*) to identify and characterize cellular processes that regulate skeletal muscle function. The mouse model is also utilized to develop various pre-clinical models of myopathies and to assess the efficacy of therapeutic interventions (Frontera and Ochala, 2015). Several different *in vivo* and *ex vivo* approaches can be employed to assess skeletal muscle force production, each with their own unique advantages and disadvantages. Examples of *in vivo* tests include muscle torque and grip strength (Iyer et al., 2016). Most *in vivo* assessments are not terminal and allow for longitudinal measures of force production while retaining muscle perfusion and neural function. However, the *in vivo* approach is limited to testing muscle groups rather than assessing individual muscles and is technically more challenging. In contrast, the *ex vivo* approach allows for more precise control of the environment of the muscle and accurate measure of maximum force-producing capacity. *Ex vivo* testing provides the investigator the ability to assess the inherent strength of the muscle independent of other variables (e.g., blood flow, nerve conduction, etc.) that could affect force production *in vivo*. Limitations of the *ex vivo* approach include muscle size (only small muscles can be assessed), a limited ability of the tissue bath to mimic the *in vivo* environment, and the inability to assess any dysfunction that develops in the neuromuscular system.

We surveyed the literature to identify published studies that assessed *ex vivo* isometric force output in murine EDL and soleus skeletal muscles, which revealed a wide degree of variation across reported values (summarized in Tables 1 and 2). The reasons for the variation are likely multi-factorial and may include technique driven explanations, such as surgical precision and equipment/protocol specifications. A limitation to accurate comparison of the data presented in the included publications is the lack of reported parameters for contractile analysis protocols. Stimulation frequency, train duration, and voltage are among the most influential factors of force production during *ex vivo* contractile assessment. These parameters are variable throughout the literature and unfortunately are not always specified by authors. However, a portion of the differences observed in the literature may also be explained by intrinsic characteristics of the mouse strain. Muscle fiber type composition, for example, is a key determinant of contraction. Not only are different muscles (e.g., EDL vs. soleus) composed of different fiber type proportions, it is likely that fiber type distributions vary within the same muscle of different mouse strains.

**TABLE 1:** Reported values of absolute (P_o_) and specific (sP_o_) force production from the EDL muscle of various strains (NR = value either absent or not reported in N/cm^2^; ∼ = value estimated from article figures). PubMed and Google Scholar searches included multiple different combinations of the following key terms—‘mouse,’ ‘murine,’ ‘skeletal muscle,’ ‘contraction,’ ‘force,’ ‘C57BL/6,’ ‘C57BL/6J,’ ‘C57BL/6NJ,’ ‘BALB/cJ,’ ‘C57BL/10,’ ‘FVB/NJ.’

Reference	Strain	Sex	Age (weeks)	P_o_ (mN)	sP_o_ (N/cm^2^)
Amthor, et al. (2007). *PNAS*, *104* (6), 1835–1840	C57BL/6	M	8	166 ± 1	NR
Barton, E. R. (2010). *JBC*, *285* (22), 17263–17270	C57BL/6	both	7 to 8	258 ± 45	20.4 ± 2.4
Barton et al. (2005). *Muscle & Nerve, 32* (6), 751–760	C57BL/6	M	8	400.5 ± 14.9	22.6 ± 0.88
Brooks & Faulkner, (1988). *J Physiol*, *404* (1), 71–82	C57BL/6	M	8 to 12	413 ± 11	23 ± 0.8
Gomez-Cabrera et al. (2010). *Am J Physiol-Regul Integr Comp Physiol*, *298* (1), R2-R8	C57BL/6	M	12	381.0 ± 51	NR
Gong et al. (2003). *Am J Physiol-Cell Physiol*, *285* (6), C1464-C1,474	C57BL/6	NR	NR	NR	32.3 ± 3.4
Graber et al. (2018). *Exp Gerontol*, *106*, 88–100	C57BL/6	M	28	418.3	57.3
Moorwood & Barton (2014). *Hum Mol Genet*, *23* (20), 5,325–5,341	C57BL/6	M	22	406 ± 20.1	∼23
Moran et al. (2006). *J Appl Physiol*, *100* (2), 548–559	C57BL/6	F	34	376 ± 26	NR
Schmidt et al. (2017). *J Vasc Surg*, *65* (5), 1,504–1,514	C57BL/6	M	12 to 16	NR	∼75
Smith & Barton (2014). *Am J Physiol-Cell Physiol*, *306* (10), C889-C898	C57	M	20	351 ± 35	20.4 ± 2.1
Dufresne et al. (2015). *Am J Pathol*, *185* (4), 920–926	C57BL/10ScSnJ	M	5	NR	∼14
Dufresne et al. (2016). *Am J Physiol-Cell Physiol*, *310* (8), C663-C672	C57BL/10J	M	12 to 18	328.5 ± 16.7	∼18
Gehrig et al. (2008). *Exp Physiol*, *93* (11), 1,190–1,198	C57BL/10ScSn	M	8 to 10	392.6 ± 13.7	26.37 ± 1.11
Hakim et al. (2011). J Appl Physiol, *110* (6), 1,656–1,663	BL10	M	8	NR	18.47 ± 0.53
			24	NR	18.54 ± 0.7
Hakim & Duan (2012). *Muscle & Nerve*, *45* (2), 250–256	BL10	M	24	∼410	∼19
		F	24	∼390	20.5 ± 0.9
Hamoudi et al. (2019). *Hum Mol Genet*, *28* (18), 3,101–3,112	C57BL/10ScSnJ	M	20	386.4 ± 12.7	∼21
Harcourt et al. (2005). Am J Pathol, *166* (4), 1,131–1,141	C57BL/10ScSn	M	8	312 ± 4	21.5 ± 0.3
			9	355 ± 17	20.4 ± 0.5
Millay et al. (2008). *Nat Med*, *14* (4), 442–447	C57BL/10	both	10	NR	∼22
Wasala et al. (2015). *PLoS Curr*, *7*	BL10	M	8	NR	18.54 ± 0.57
Widrick et al. (2011). *Muscle & Nerve*, *44* (4), 563–570	C57BL/10SnJ	M	10	NR	23.7 ± 1.3
Goldberg et al. (2019). *Front Physiol*, *10*, 804	BALB/cJ	M	12 to 18	364	∼15
Regan et al. (2017). *Front Endocrinol*, *8*, 358	BALB/c	F	5	NR	∼43
Schmidt et al. (2018). *Am J Pathol*, *188* (5), 1,246–1,262	BALB/cJ	M	12 to 16	NR	∼13
Schmidt et al. (2017). *J Vasc Surg*, *65* (5), 1,504–1,514	BALB/cJ	M	12 to 16	NR	∼100
Schmidt et al. (2020). *PloS One*, *15* (4), e0225922	BALB/c	M	16 to 24	NR	∼14
Ammar et al. (2015). *J Gen Physiol*, *146* (6), 509–525	FVB	NR	8 to 12	NR	∼30
Chaillou et al. (2017). *Physiol Rep*, *5* (11), e13261	FVB/NRj	F	8 to 11	∼285	∼ 55
Pierno et al. (2013). *PLoS One*, *8* (6)	FVB	NR	24	NR	∼11
Wasala et al. (2015). *PLoS Curr*, *7*	FVB/NJ	M	12	NR	18.9 ± 0.87

**TABLE 2:** Reported values of absolute (P_o_) and specific (sP_o_) force production from the soleus muscle of BL6, BL10, BALB/c, and FVB strains (NR = value either absent or not reported in N/cm^2^; ∼ = value estimated from article figures). PubMed and Google Scholar searches included multiple different combinations of the following key terms—‘mouse,’ ‘murine,’ ‘skeletal muscle,’ ‘contraction,’ ‘force,’ ‘C57BL/6,’ ‘C57BL/6J,’ ‘C57BL/6NJ,’ ‘BALB/cJ,’ ‘C57BL/10,’ ‘FVB/NJ.’

Reference	Strain	Sex	Age (weeks)	P_o_ (mN)	sP_o_ (N/cm^2^)
Axell et al. (2006). *Am J Physiol-Endocrinol Metab*, *291* (3), E506-E516	C57BL/6	M	18	∼250	25.87 ± 0.76
Baumann et al. (2016). *PloS One*, *11* (8)	C57BL/6	M	26	215.28 ± 15.57	24.1 ± 1.94
Brooks & Faulkner, (1988). *J Physiol*, *404* (1), 71–82	C57BL/6	M	8 to 12	213 ± 6	20.6 ± 0.67
Gomez-Cabrera et al. (2010). *Am J Physiol-Regul Integr Comp Physiol*, *298* (1), R2-R8	C57BL/6	M	12	247 ± 39	NR
Gong et al. (2003). *Am J Physiol-Cell Physiol*, *285* (6), C1464-C1,474	C57BL/6J	NR	NR	NR	28.9 ± 1.9
Graber et al. (2018). *Exp Gerontol*, *106*, 88–100	C57BL/6	M	20	233.8	26
Houngbédji et al. (2009). *Microbes infect*, *11* (2), 238–244	C57BL/6	M	NR	245.8 ± 8	24.6 ± 1.7
Moran et al. (2006). *J Appl Physiol*, *100* (2), 548–559	C57BL/6	F	34	164 ± 15	NR
Smith & Barton (2014). *Am J Physiol-Cell Physiol*, *306* (10), C889-C898	C57	M	20	184 ± 23	17.0 ± 1.9
Dufresne et al. (2015). *Am J Pathol*, *185* (4), 920–926	C57BL/10ScSn	M	5	NR	∼14.5
Dufresne et al. (2016). *Am J Physiol-Cell Physiol*, *310* (8), C663-C672	C57BL/10J	M	12 to 18	260.9 ± 11.8	∼22
Gehrig et al. (2008). *Exp Physiol*, *93* (11), 1,190–1,198	C57BL/10ScSn	M	8 to 10	235.7 ± 8.5	25.11 ± 0.75
Gregorevic et al. (2004). *Muscle & Nerve*, *30* (3), 295–304	C57BL/10ScSn	M	5 to 6	211.1 ± 6.4	22.0 ± 0.96
Hamoudi et al. (2019). *Hum Mol Genet*, *28* (18), 3,101–3,112	C57BL/10ScSnJ	M	20	268.7 ± 18.63	∼26
Moens et al. (1992). J Neurol Sci, *111* (2), 209–213	C57BL/10	NR	NR	222 ± 10	12.0 ± 0.8
Stupka et al. (2004). *Acta Neuropathologica*, *107* (4), 299–310	C57BL/10ScSn	both	5	103.6 ± 4.3	22.8 ± 0.84
Goldberg et al. (2019). *Front Physiol*, *10*, 804	BALB/cJ	M	12 to 18	211	∼16
chmidt et al. (2020). *PloS One*, *15* (4), e0225922	BALB/c	M	16 to 24	NR	∼16
Wernig et al. (2000). *J Physiol*, *522* (2), 333–345	BALB/c	F	12 to 24	176 ± 13	NR
Ammar et al. (2015). *J Gen Physiol*, *146* (6), 509–525	FVB	NR	8 to 12	NR	∼19
Chaillou et al. (2017). *Physiol Rep*, *5* (11), e13261	FVB/NRj	F	8 to 11	∼135	∼40
Pierno et al. (2013). *PLoS One*, *8* (6)	FVB	NR	24	NR	∼17

Advancements in genetic engineering over past decades have facilitated an increased use of genetically modified mice for biomedical research. Investigators are now able to modify the mouse germline through a variety of approaches that result in the generation of transgenic or knockout mice with distinct physiological characteristics (Brooks Mobley et al., 2020). With the development of new models, the range of different mouse strains being employed by investigators is growing. An increase in the number of inbred strains alone has led to extensive documentation of genomic variability that associates with functional discrepancies across research in both health and disease (Yalcin et al., 2011; Doran et al., 2016; Li et al., 2018). Many previous studies have shown strain differences in numerous physiological measures or differential responses to the same physiological insults, indicating that consideration of mouse strain is an important aspect of experiment design (Coleman and Hummel, 1973; Beckwith et al., 2005; Fisher-Wellman et al., 2016; Schmidt et al., 2018). The leveraging of strain differences as a means to study complex biological traits or disease phenotypes was recently highlighted by Lusis et al. (2016) using the Hybrid Mouse Diversity Panel (HMDP). The panel is made up of approximately 100 different strains of mice that various investigations have used to identify genes that influence a number of biological traits, such as bone mineral density and heart rate (Lusis et al., 2016). When examining publications that employ the HMDP, it is clear that some strains show remarkable similarity depending upon the experimental outcome measure, while other strains exhibit a range of differences that have physiological significance (Davis et al., 2013; Parks et al., 2015; Rau et al., 2015). However, skeletal muscle literature lacks documentation of possible differences in common measures of muscle contractile function across different mouse strains.

The purpose of the present study is to assess if differences in key contractile parameters of the EDL and soleus muscles exist in common laboratory mouse strains. Considering the notable variation in reported values of murine *ex vivo* force production, we hypothesized that mice with different genetic backgrounds exhibit measurable distinctions in one or more contractile properties of the EDL and/or the soleus muscle.

## Materials and methods

### Animals

Male mice on the C57BL/6NJ, BALB/cJ, FVB/NJ, C57BL/6J, and C57BL/10 backgrounds (*n* = 6 per group) were obtained from Jackson Laboratories at 6–8 weeks of age. Although the mice were not fully matured, this age range was chosen to establish baseline phenotypes of each strain as they enter adulthood. Mice were housed in ventilated cages within a temperature- (22°C) and light- (12/12 h light/dark) controlled facility with access to food and water *ad libitum*. Mice were anesthetized *via* isoflurane and euthanized via cervical dislocation prior to tissue removal (described below). All animal procedures were approved by the Institutional Review Committee at East Carolina University and complied with the Guide for the Care and Use of Laboratory Animals, Institute of Laboratory Animal Resources, Commission on Life Sciences, National Research Council.

### *Ex vivo* isometric contractile analysis

Isometric force production and fatigue resistance of the EDL and soleus muscles were assessed *ex vivo* as described previously (Tarpey, et al., 2019a). Muscles were surgically excised and mounted to a force transducer apparatus (Aurora Scientific Inc., 150A) *via* 4-0 silk suture ligatures tied at each tendon. Muscles were suspended in a bath of oxygenated Krebs Ringer Buffer (KRB—[mM] *119 NaCl, 5.0 KCl, 5.0 NaHCO*_*3*_ *, 1.25 CaCl*_*2*_ *, 1.0 KH*_*2*_ *PO*_*4*_ *, 10 HEPES, 1.0 MgSO*_*4*_; pH 7.2) maintained at room temperature (22–23°C). Following a 10-min equilibration period, optimal resting length (L_0_) was established by subjecting the muscles to a single 1 Hz twitch stimulation every 30 s and adjusting muscle length to determine maximal force output. Supramaximal simulations were induced by a bi-phasic stimulator (Aurora Scientific, 701C) and delivered using parallel platinum electrodes (separated by ∼9 mm) that flanked the muscle in the bath solution. Following determination of L_0_, muscles were stimulated every 60 s at 10, 20, 40, 60, 80, 100, and 120 Hz with 200 ms trains (0.2 ms pulse width, 40 V) to generate force-frequency curves. Muscles rested for an additional 60 s prior to undergoing a 5-min fatigue resistance protocol (100 Hz every 2 s, 150 contractions using the above parameters). Following measurement of L_0_ with digital calipers, proximal and distal tendons were trimmed using microscissors and muscles were blotted to remove any excess KRB prior to measuring muscle wet weight. Force output was collected and analyzed using Dynamic Muscle Control LabBook 610A and Dynamic Muscle Analysis 611A software, respectively (Aurora Scientific Inc.,). Using the same software, half-relaxation time (½ RT) data were collected from the 100 Hz contractions of each muscle. Absolute force (mN) data were normalized to muscle mass and physiological cross-sectional area (PCSA) to determine muscle specific force (N/cm^2^). PCSA was estimated mathematically using previously outlined equations, which account for the density of mammalian skeletal muscle (1.06 g/cm^3^; Mendez and Keys, 1960) and myofiber length/whole muscle length ratios of 0.45 and 0.7 for the EDL and soleus, respectively (Brooks and Faulkner, 1988).

### Immunofluorescent analysis of myofiber cross-sectional area and fiber type composition

Myofiber CSA of the EDL and soleus was measured as described previously (Tarpey, et al*.*, 2019b). Briefly, muscles were embedded in optimal cutting temperature (OCT) medium and frozen in ice-cold isopentane. Cryosections (10 μm) taken from the muscle midbelly were probed with the following primary antibodies - dystrophin (RB-9024, 1:100 Thermo Fisher), myosin heavy chain (MHC) Type I (DSHB-#BA-F8, 1:50), MHC Type IIa (DSHB-#SC-71, 1:50), and MHC Type IIb (DSHB-#BF-F3, 1:50). The following secondary antibodies were used at 1:250—dystrophin (A-21244; Thermo Fisher), MHC Type I Alexa Fluor 350 (#A-21140, Life Technologies), MHC Type IIa Alexa Fluor 488 (#A-21121, Life Technologies), and MHC Type IIb Alexa Fluor 546 (#A-21045, Life Technologies). Type IIx fibers remained unstained. Cross-sections were imaged using an EVOS FL auto microscope (Life Technologies) and analyzed using ImageJ software (CSA—*n* = ≥500 myofibers per muscle; fiber typing—*n* = ≥300 myofibers per muscle; *n* = 5 muscles per strain with exception to BL10 fiber typing—*n* = 4).

### Statistical analysis

Data in each group were statistically compared using GraphPad Prism 9.0 software (DotMatics). Gaussian distribution of data was confirmed using multiple normality tests (Anderson-Darling, D’Agostino-Pearson, Shapiro-Wilk, and Kolmogorov-Smirnov). ANOVA with Tukey’s *post hoc* analysis and coefficient of variation tests were employed as appropriate. Statistical significance was set at *p* ≤ 0.05. All data are presented as mean ± SEM.

## Results

### Absolute force

**FIGURE 1:**
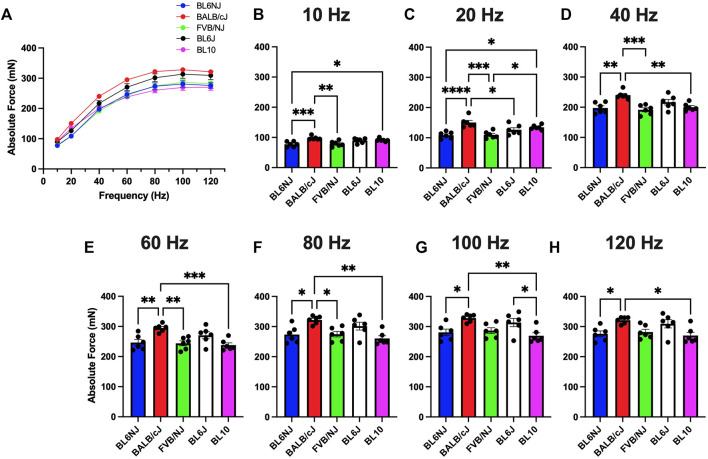
**(A–H)**. Absolute isometric force production of the EDL muscle from various strains of mice. **(A)** Force-frequency curves (Blue = BL6NJ, Red = BALB/cJ, Green = FVB/NJ, Black = BL6J, Pink = BL10), **(B)** force produced at 10 Hz, **(C)** force produced at 20 Hz, **(D)** force produced at 40 Hz, **(E)** force produced at 60 Hz, **(F)** force produced at 80 Hz, **(G)** force produced at 100 Hz, **(H)** force produced at 120 Hz. Statistically significant difference is indicated by one symbol = *p* < 0.05; two symbols = *p* < 0.005; three symbols = *p* < 0.001; four symbols = *p* < 0.0001.

EDL. Assessment of absolute force production (P_o_) in the EDL muscle revealed some variation across all the strains (Figures 1A–H). Across majority of the stimulation frequencies, the BALB/c EDL muscles produced significantly more P_o_ than the other strains with exception to the BL6J and the BL10 mice at only the lower frequencies (10 and 20 Hz, Figure 1C). EDLs from BL10 mice achieved the lowest average maximal P_o_ (270 ± 11 mN) at 120 Hz (Figure 1H) whereas the average maximal P_o_ was highest in BALB/cJ EDLs (328.1 ± 6 mN) at 100 Hz (Figure 1G).

**FIGURE 2:**
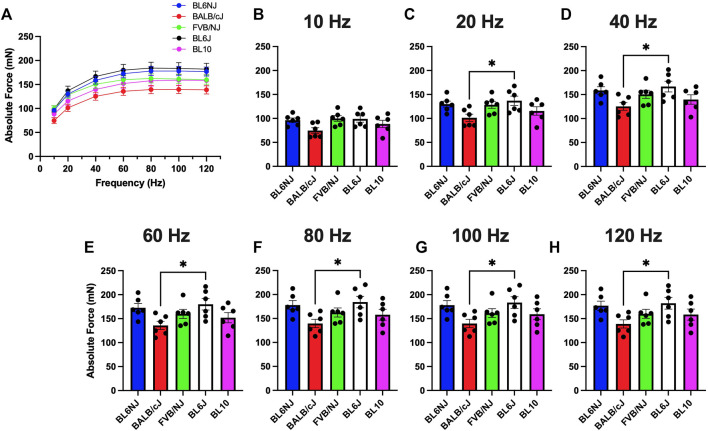
**(A–H)**. Absolute isometric force production of the soleus muscle from various strains of mice. **(A)** Force-frequency curves (Blue = BL6NJ, Red = BALB/cJ, Green = FVB/NJ, Black = BL6J, Pink = BL10), **(B)** force produced at 10 Hz, **(C)** force produced at 20 Hz, **(D)** force produced at 40 Hz, **(E)** force produced at 60 Hz, **(F)** force produced at 80 Hz, **(G)** force produced at 100 Hz, **(H)** force produced at 120 Hz. Statistically significant difference is indicated by one symbol = *p* < 0.05.

Soleus*.* In contrast to the EDL, BALB/cJ soleus muscles demonstrated the lowest maximal P_o_ across strains (139.7 ± 9 mN) at 100 Hz (Figure 2G), with significant differences between BL6J mice at all frequencies except 10 Hz (Figures 2C–H). Soleus P_o_ in the other strains were similar across the frequencies of stimulation (Figures 2A–H).

### Specific force

**FIGURE 3:**
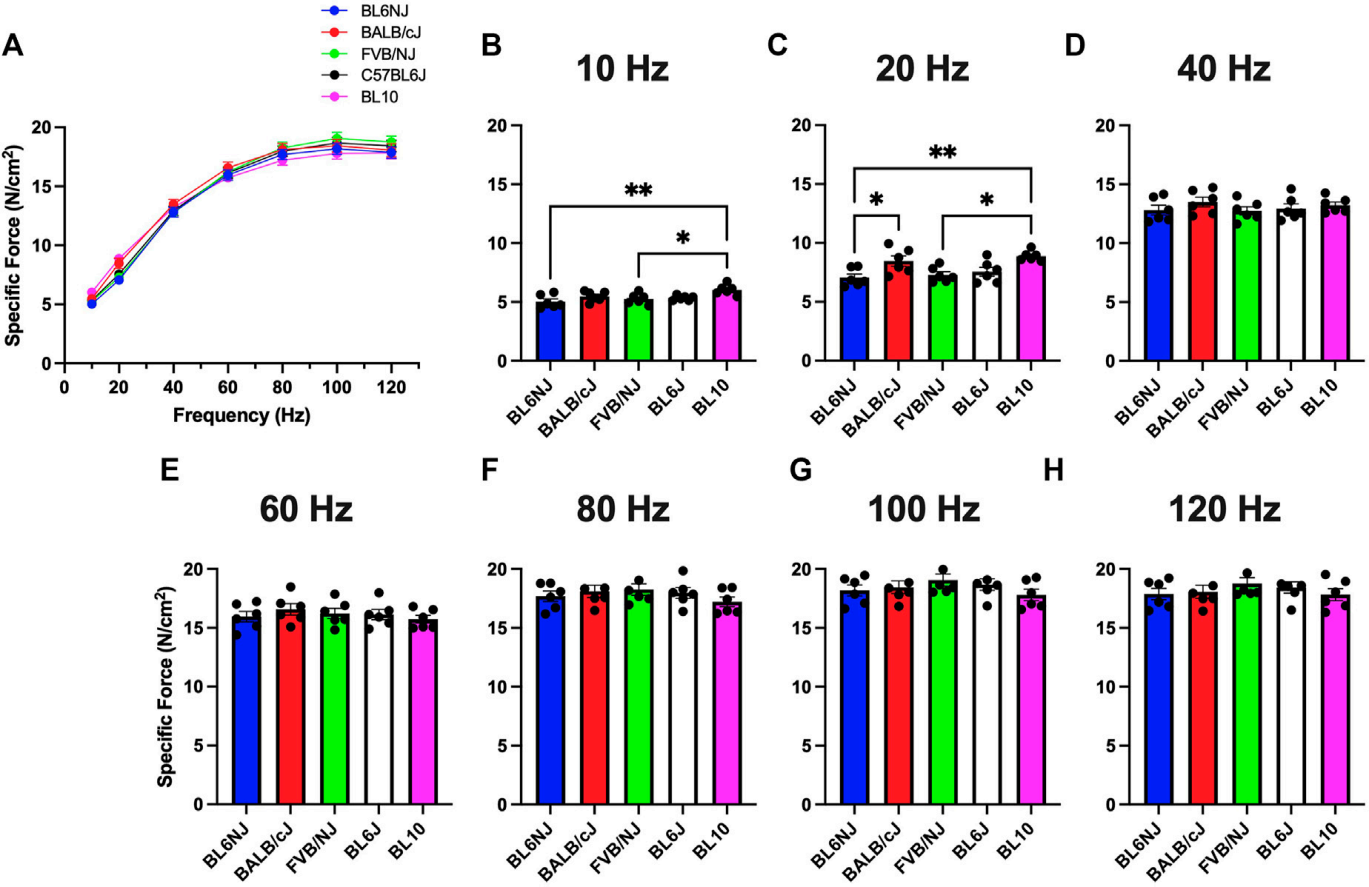
**(A–H)**. Specific isometric force production of the EDL muscle from various strains of mice. **(A)** Force-frequency curves (Blue = BL6NJ, Red = BALB/cJ, Green = FVB/NJ, Black = BL6J, Pink = BL10), **(B)** force produced at 10 Hz, **(C)** force produced at 20 Hz, **(D)** force produced at 40 Hz, **(E)** force produced at 60 Hz, **(F)** force produced at 80 Hz, **(G)** force produced at 100 Hz, **(H)** force produced at 120 Hz. Statistically significant difference is indicated by one symbol = *p* < 0.05; two symbols = *p* < 0.005.

EDL ***.*** Muscle specific force (sP_o_) was calculated by normalizing absolute force production values to muscle size as previously described (Tarpey, et al., 2019a) Very few differences were detected in EDL sP_o_ across strains with exception to the lower frequencies of stimulation (Figures 3A–H). At 10 Hz, BL10 mice showed higher sP_o_ than BL6NJ (*p* = 0.004) and FVB/NJ (*p* = 0.036) mice (Figure 3B). This relationship was also observed at 20 Hz (BL10 vs. BL6NJ *p* = 0.004; BL10 vs. FVB/NJ *p* = 0.016) in addition to BL10 EDLs producing higher sP_o_ than BL6J mice (*p* = 0.037).

**FIGURE 4:**
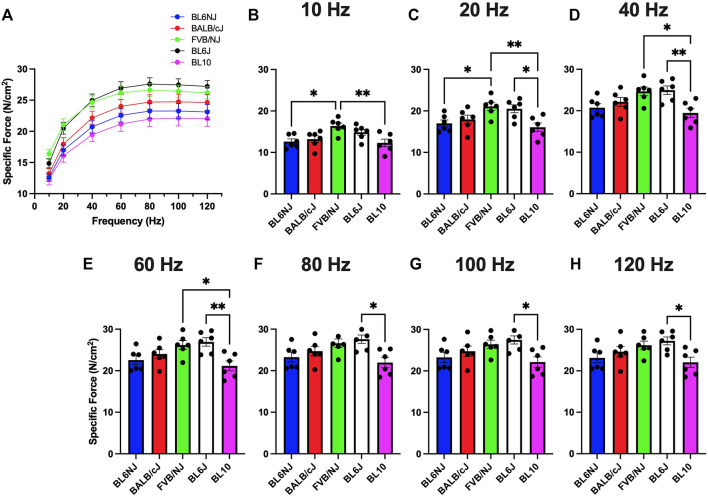
**(A–H)**. Specific isometric force production of the soleus muscle from various strains of mice. **(A)** Force-frequency curves (Blue = BL6NJ, Red = BALB/cJ, Green = FVB/NJ, Black = BL6J, Pink = BL10), **(B)** force produced at 10 Hz, **(C)** force produced at 20 Hz, **(D)** force produced at 40 Hz, **(E)** force produced at 60 Hz, **(F)** force produced at 80 Hz, **(G)** force produced at 100 Hz, **(H)** force produced at 120 Hz. Statistically significant difference is indicated by one symbol = *p* < 0.05; two symbols = *p* < 0.005.

Soleus. The soleus muscles from the BL10 mice exhibited significantly lower sP_o_ as compared to BL6J mice at all frequencies with exception to 100 Hz (Figures 4A–H), while only different than FVB/NJ at lower frequencies (10–60 Hz, Figures 4B–E). FVB/NJ soleus muscles also produced greater sP_o_ than those of BL6NJ mice at 10 and 20 Hz (Figures 4B,C).

### Fatigue susceptibility

**FIGURE 5:**
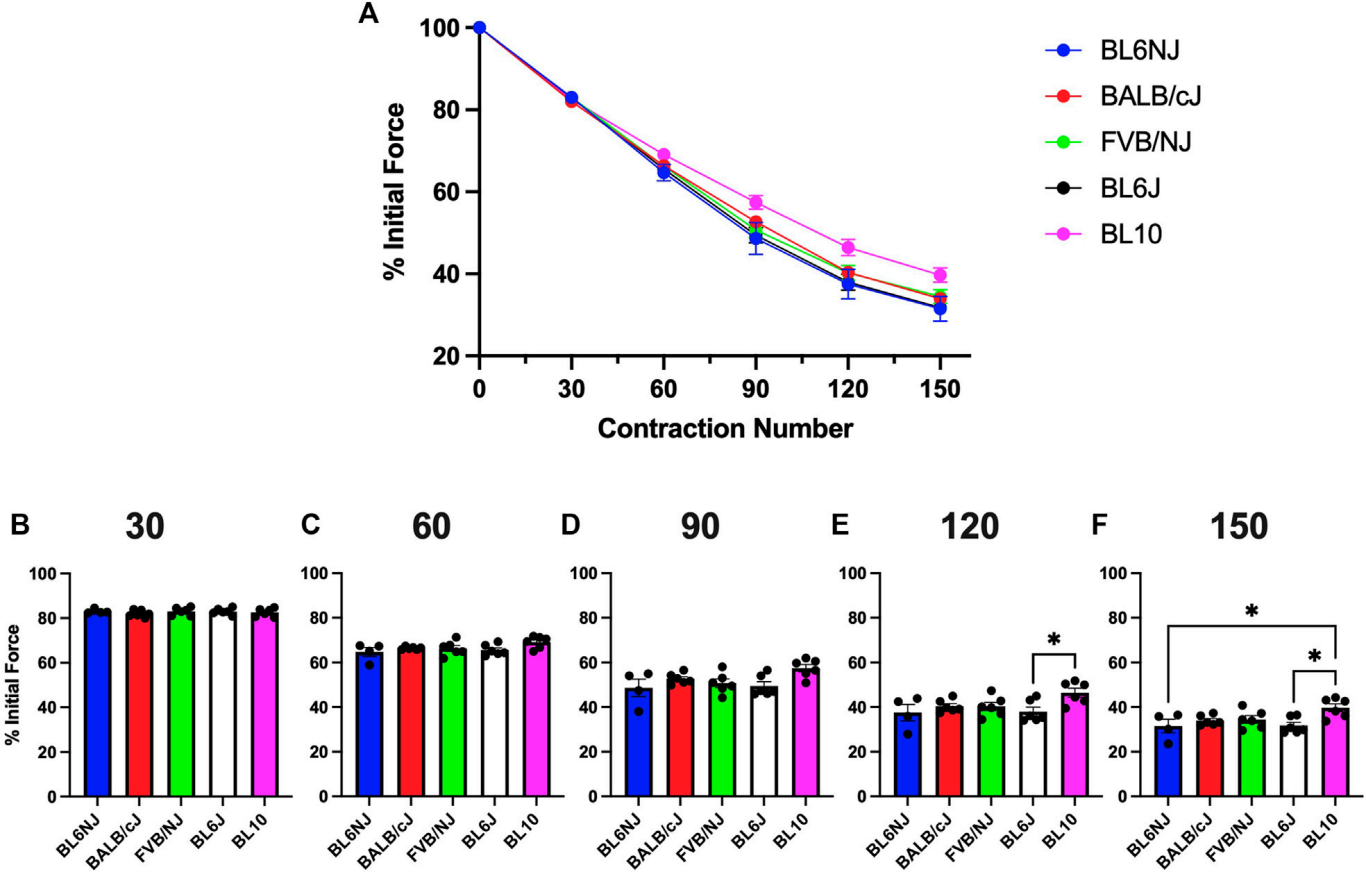
**(A–F)**. Isometric fatigue of the EDL muscle from various strains of mice. **(A)** Fatigue curve (Blue = BL6NJ, Red = BALB/cJ, Green = FVB/NJ, Black = BL6J, Pink = BL10), **(B)** percent initial force produced after 30 contractions, **(C)** percent initial force produced after 60 contractions, **(D)** percent initial force produced after 90 contractions, **(E)** percent initial force produced after 120 contractions, **(F)** percent initial force produced after 150 contractions. Statistically significant difference is indicated by one symbol = *p* < 0.05.

EDL. No significant differences existed in EDL fatigue susceptibility across strains until after 120 contractions (Figures 5A–D). BL10 EDLs maintained a significantly higher percentage of initial force produced compared to BL6J EDLs after 120 (*p* = 0.042) and 150 contractions (*p* = 0.019) as well as compared to BL6NJ EDLs after 150 contractions (*p* = 0.033; Figures 5E,F).

**FIGURE 6:**
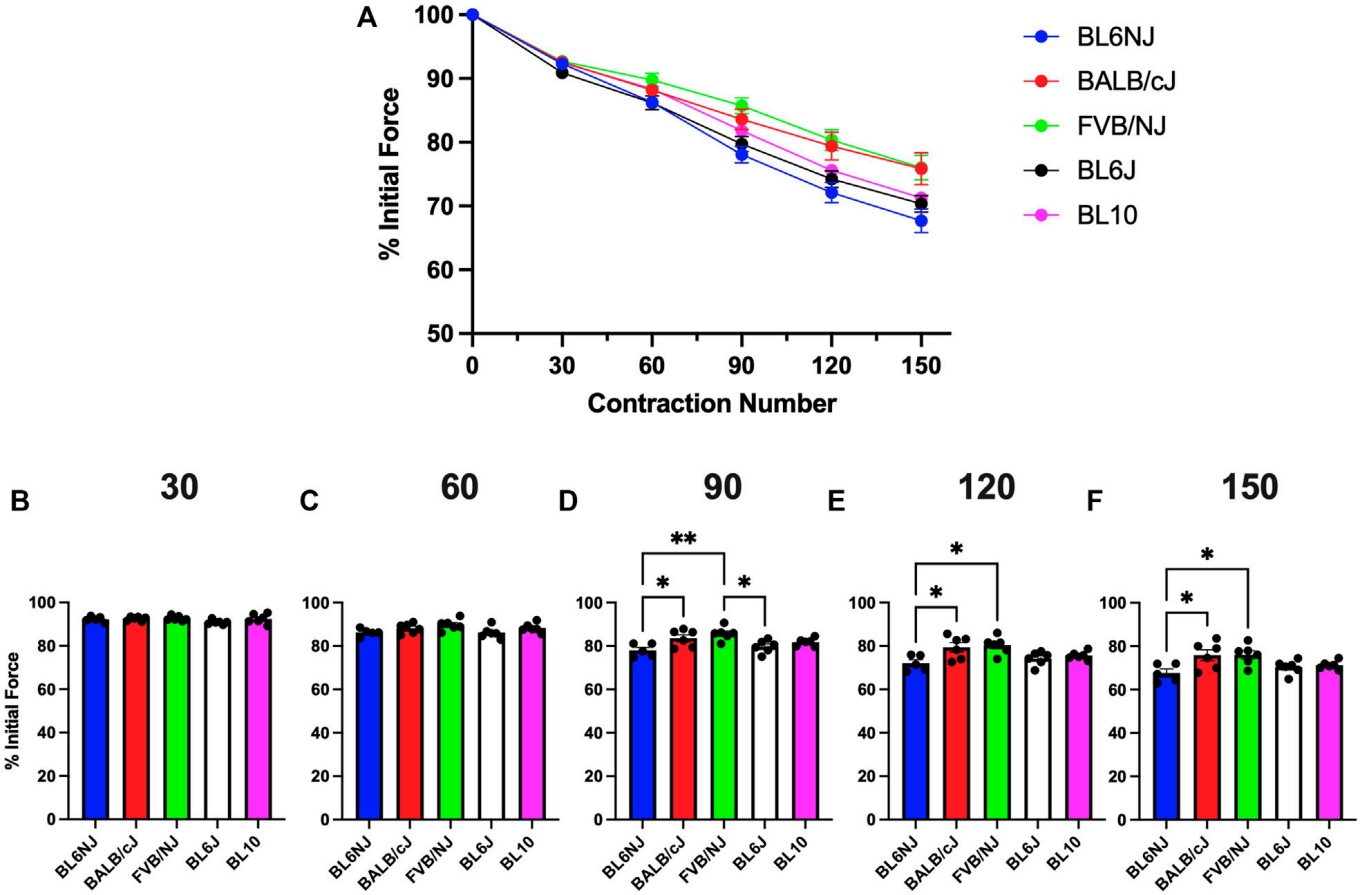
**(A–F)**. Isometric fatigue of the soleus muscle from various strains of mice. **(A)** Fatigue curve (Blue = BL6NJ, Red = BALB/cJ, Green = FVB/NJ, Black = BL6J, Pink = BL10), **(B)** percent initial force produced after 30 contractions, **(C)** percent initial force produced after 60 contractions, **(D)** percent initial force produced after 90 contractions, **(E)** percent initial force produced after 120 contractions, **(F)** percent initial force produced after 150 contractions. Statistically significant difference is indicated by one symbol = *p* < 0.05; two symbols = *p* < 0.005.

Soleus. The soleus from the BL6NJ mice exhibited greater fatigue susceptibility as compared to BALB/cJ and FVB/NJ mice after 90–150 contractions (Figures 6A–F). The soleus muscles from the BL6/J mice appear to exhibit a similar phenotype as the BL6NJ soleus when compared to FVB/NJ mice, however statistical significance was only achieved after 90 contractions (*p* = 0.018) and not at any other point (Figure 6D).

### Half-relaxation time

**FIGURE 7:**
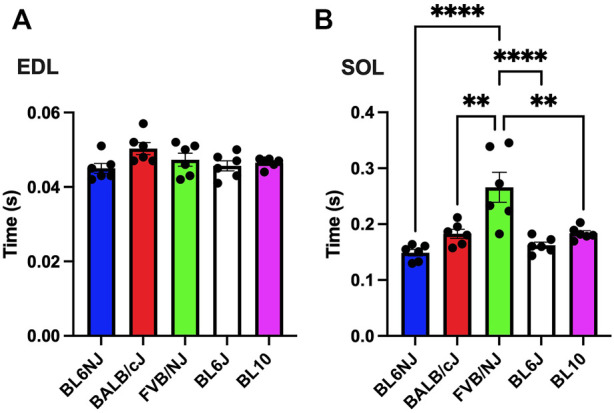
**(A,B).** Time to ½ relaxation (½ RT) in the EDL **(A)** and soleus muscle **(B)** from various strains of mice. Times were captured at the 100 Hz contraction. Statistically significant difference is indicated by one symbol = *p* < 0.05; two symbols = *p* < 0.005; three symbols = *p* < 0.001; four symbols = *p* < 0.0001.

EDL. No significant differences were found in ½ RT of the EDL muscle across strains (Figure 7A).

Soleus. Analysis of ½ RT revealed a significantly longer time for relaxation of the FVB/NJ soleus as compared all other strains (vs. BL6NJ *p* < 0.0001; vs. BALB/cJ *p* = 0.001; vs. BL6J *p* = 0.0001; vs. BL10 *p* = 0.0002, Figure 7B).

### Muscle mass and optimal length

**FIGURE 8:**
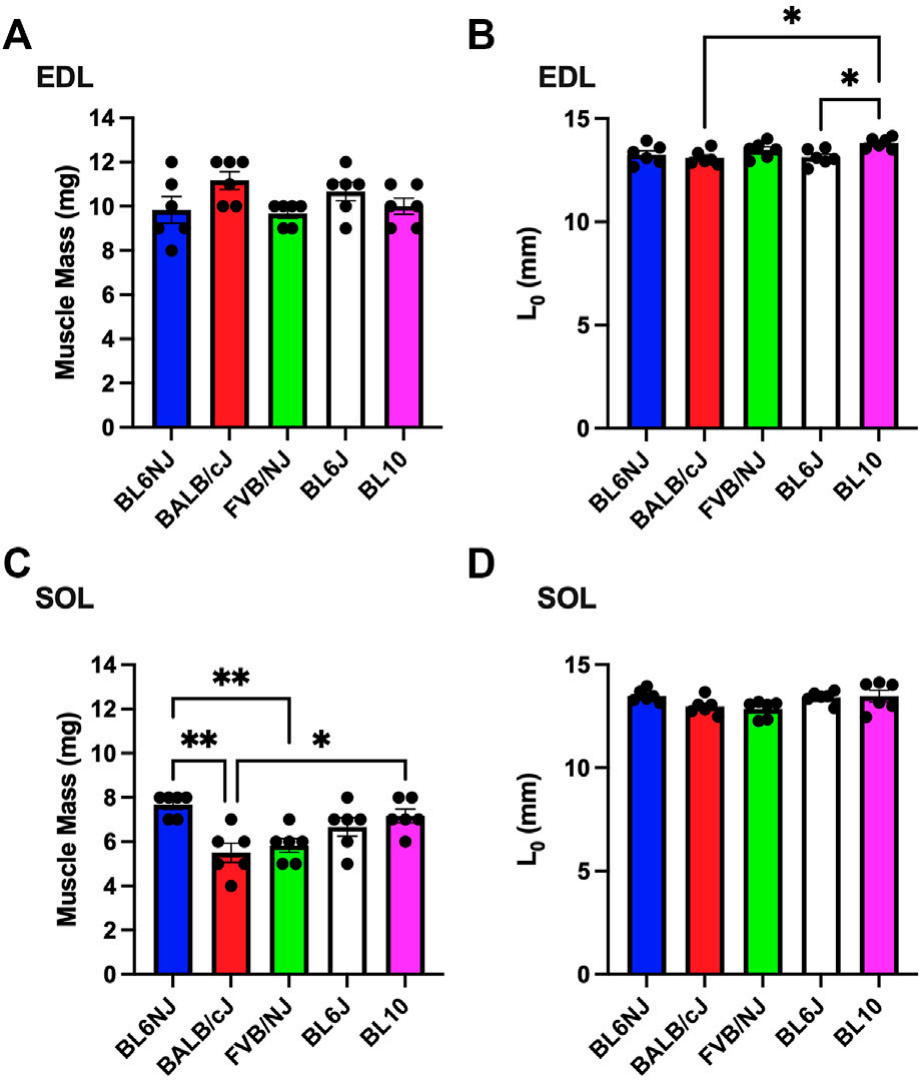
**(A**–**D)**. Muscle mass and optimal muscle length of the EDL (A,B) and soleus muscle (C,D) from various strains of mice. Statistically significant difference is indicated by one symbol = *p* < 0.05; two symbols = *p* < 0.005.

EDL. No significant differences existed in muscle mass of the EDL across strains (Figure 8A). Optimal lengths of BL10 EDLs were significantly different compared to the BALB/cJ (*p* = 0.022) and BL6J (*p* = 0.028) muscles (Figure 8B).

Soleus. Soleus muscle mass was significantly greater in BL6NJ mice as compared to BALB/cJ (*p* = 0.001) and FVB/NJ (*p* = 0.007) mice (Figure 8C). BL10 soleus muscle mass was significantly greater than that of BALB/cJ mice (*p* = 0.017, Figure 8C). No differences in soleus optimal lengths were observed across strains (Figure 8D).

### Myofiber cross-sectional area

**FIGURE 9:**
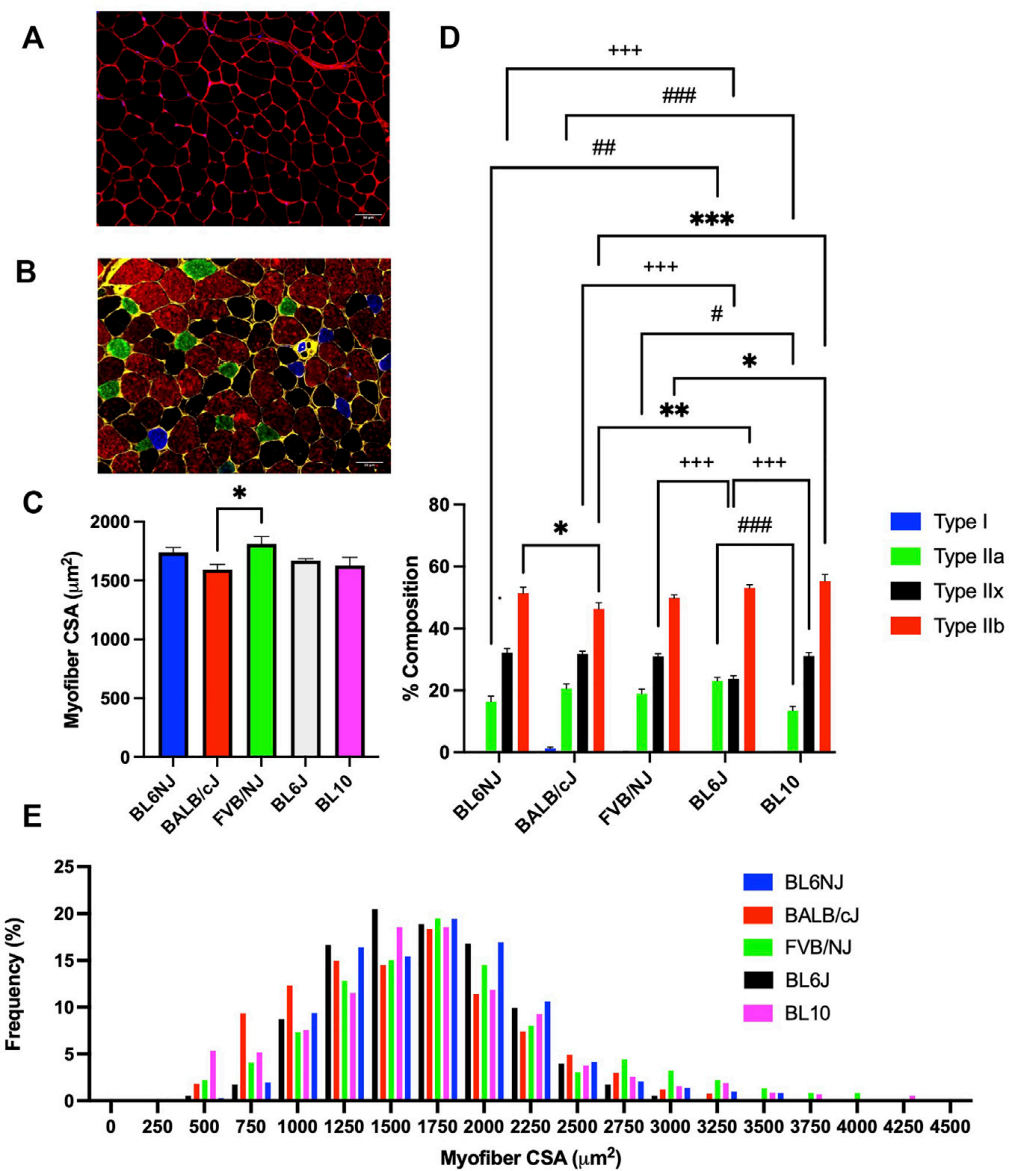
Average myofiber CSA and fiber type composition of the EDL muscle from various strains of mice. **(A)** Representative cross-sectional image of a BL10 EDL stained for dystrophin to outline the sarcolemma (red) and nuclei (DAPI, blue); **(B)** Representative cross-sectional image of a BL6NJ EDL probed for dystrophin (yellow) and the following MHC isoforms—Type I (blue), Type IIa (green), Type IIx (black), and Type IIb (red); **(C)** Average CSA of individual fibers; **(D)** Average fiber type distribution of individual muscles; **(E)** Frequency distribution of all fibers measured in the EDL muscles from each strain. # = difference in Type IIa; + = difference in Type IIx; * = difference in Type IIb. Statistically significant difference is indicated by one symbol = *p* < 0.05; two symbols = *p* < 0.005; three symbols = *p* < 0.001.

EDL. The average CSA of EDL myofibers from FVB/NJ mice (1811 ± 62.91 µm^2^) was significantly greater than BALB/cJ EDL myofibers (1593 ± 43.37 µm^2^, *p* = 0.044, Figure 9C). No other significant differences in average myofiber CSA of the EDL were demonstrated across strains. Assessment of individual myofiber CSA frequency distribution revealed a leftward shift of the distribution in the BALB/cJ EDLs (Figure 9E). Individual CSA frequency distributions from the EDLs of each strain are presented in Supplementary Figure S1 (Supplementary Materials).

**FIGURE 10:**
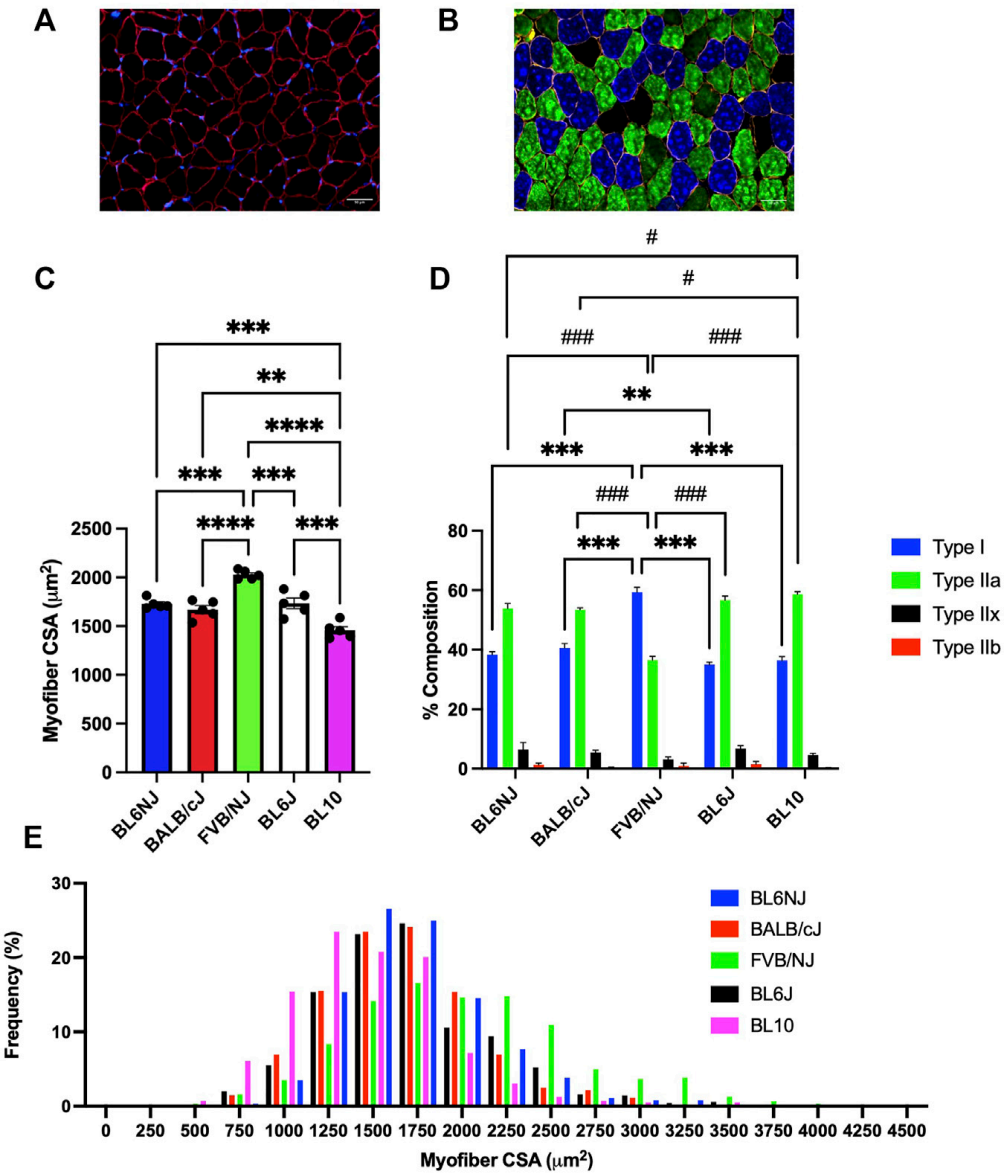
Average myofiber CSA and fiber type composition of the soleus muscle from various strains of mice. **(A)** Representative cross-sectional image of a BL6J soleus stained for dystrophin to outline the sarcolemma (red) and nuclei (DAPI, blue); **(B)** Representative cross-sectional image of a BALB/cJ soleus probed for dystrophin (yellow) and the following MHC isoforms—Type I (blue), Type IIa (green), Type IIx (black), and Type IIb (red); **(C)** Average CSA of individual fibers; **(D)** Average fiber type distribution of individual muscles; **(E)** Frequency distribution of all fibers measured in the soleus muscles from each strain. * = difference in Type I; # = difference in Type IIa. Statistically significant difference is indicated by one symbol = *p* < 0.05; two symbols = *p* < 0.005; three symbols = *p* < 0.001.

Soleus ***.*** The FVB soleus muscles contained significantly larger myofibers than all other strains (vs. BL6NJ *p* = 0.0001; BALB/cJ *p* < 0.0001; BL6J *p* = 0.0002; vs. BL10 *p* < 0.0001; Figure 10C). BL10 soleus myofiber CSA was significantly smaller than that of all other strains (vs. BL6NJ *p* = 0.001; vs. BALB/cJ *p* = 0.01; vs. BL6J *p* = 0.0004; Figure 10C). The myofiber CSA frequency distribution revealed a rightward shift in the FVB/NJ soleus and a leftward shift in the BL10 soleus (Figure 10E). Individual CSA frequency distributions from the soleus muscles of each strain are presented in Supplementary Figure S2 (Supplementary Mateials).

### Fiber type composition

EDL. BL10 EDLs demonstrated a significantly lower percentage of Type IIa fibers as compared to BL6J (*p* < 0.0001), BALB/cJ (*p* = 0.001), and FVB/NJ (*p* = 0.019) EDLs (Figure 9D). BL6J EDLs contained significantly more Type IIa fibers than BL6NJ mice (*p* = 0.002, Figure 9D), but demonstrated the lowest percentage of Type IIx fibers as compared to all other strains (vs. BL6NJ and BALB/cJ *p* < 0.001; vs. FVB/NJ *p* = 0.001; vs. BL10 *p* = 0.0005, Figure 9D). Finally, analysis of Type IIb fibers revealed a significantly greater percentage in BL10 EDLs as compared to the FVB/NJ (*p* = 0.02) and BALB/cJ (*p* < 0.0001) strains (Figure 9D). BALB/cJ EDLs also contained a lower percentage of Type IIb fibers than BL6J (*p* < 0.001) and BL6NJ mice (*p* = 0.039, Figure 9D).

Soleus. The FVB mice contained the greatest percentage of Type I muscle fibers across all strains (*p* < 0.0001, Figure 10D). Type I fibers were also significantly greater in BL6J than BALB/cJ soleus muscles (*p* = 0.006, Figure 10D). Contrarily, Type IIa fibers were least abundant in the FVB/NJ soleus than those of other strains (*p* < 0.0001, Figure 10D) BL10 mice demonstrated a greater percentage of Type IIa fibers than the BL6NJ (*p* = 0.045) and BALB/cJ mice (*p* = 0.024, Figure 10D) Finally, no significant differences in the composition of Type IIx or Type IIb fibers of the soleus were observed across strains.

## Discussion

There is a long, rich history of studies assessing physiological properties of skeletal muscle using murine models. However, no consistent consideration has been given to the possibility that differences may exist between distinct strains of mice. Recently, other fields of investigation have determined that mice with differing genetic backgrounds exhibit fundamentally unique physiological properties across a variety of tissues (Schmidt et al., 2018). These distinctions have emphasized the importance of defining the mouse strain in proposed and published experimental approaches. Conceptually, this idea has not been acknowledged at the same depth in skeletal muscle, thus we sought to address this gap by characterizing various physiological properties of the EDL and soleus muscles across commonly used laboratory mouse strains.

Absolute force production of the EDL was highest in the BALB/cJ and BL6J mice, which is likely a result of greater muscle mass. Surprisingly, the soleus muscle from the BALB/cJ group exhibited the lowest absolute force and muscle mass whereas soleus absolute force was greatest in BL6NJ and BL6J mice compared to other tested strains. These data suggest that differences in absolute force production in these strains are explained by the differences in mass of the muscle.

Contrary to the observed variation in absolute force production, analysis of EDL specific force revealed largely similar results indicating that normalized force output of the EDL is similar across common laboratory strains (Figure 3). The only differences were found at lower stimulation frequencies where BL10 EDLs demonstrated greater specific force than BL6NJ and FVB/NJ mice (Figures 3B,C). However, this similarity was not demonstrated in comparing the specific force production of the soleus muscles. Across majority of the stimulation frequencies, soleus specific force was greatest in the BL6J mice and lowest in the BL10 mice (Figure 4). Specific force is calculated mathematically, which may have affected our interpretation of the data. For example, assumed ratios of myofiber length to whole muscle length may be variable in the EDL and soleus across strains, which would influence the outcome measures. Thus, future studies should assess each anatomical relationship involved in specific force calculation in each strain.

The FVB/NJ mice exhibited multiple differences in the examined muscle contractile and histological properties across strains. Myofiber CSA was largest in both the EDL and soleus of FVB/NJ mice. The FVB/NJ soleus muscles also contained the greatest proportion of Type I fibers (Figure 10D), which likely explains the greatest fatigue resistance (Figure 6) and the slowest rate of muscle relaxation (Figure 7B). BL6NJ soleus muscles were among the weakest (Figure 4) and most susceptible to fatigue (Figure 6) despite exhibiting higher muscle mass than the other strains (Figure 8D). Notably, EDL fatigue resistance was greatest in BL10 mice (Figure 5F) despite containing the lowest proportion of Type IIa myofibers (Figure 9D), which alongside Type I fibers are considered most resistant to fatigue (Fitts, 1994). Skeletal muscle fiber type, as assessed by MHC isoform prevalence, is not a precise predictor of all the physiological properties of a muscle. For example, metabolic properties can differ from the predicted phenotype when using MHC as the basis for fiber typing (Pereyra et al., 2022). It is possible that lower susceptibility to fatigue in BL10 EDLs is a result of differing metabolic regulation as compared to other strains, however this would need to be confirmed in follow up studies. Since skeletal muscle fatigue is influenced by various factors and the central nervous system is eliminated in the *ex vivo* condition, our interpretation of the present data can only be informed by the intrinsic contractile properties of the muscle. The overall similarity observed in *ex vivo* fatigue resistance of both muscles across strains may be explained similar regulation of ECC mechanisms across strains, which is possible since few differences were found in relaxation kinetics (Figure 7). Our data might suggest that SR calcium ATPase (SERCA) activity is similar across most of the strains. However, it should be noted that this study did not empirically assess ECC and is only using contractile kinetics as a predictor. Future studies are required to determine if strain-mediated differences exist in ECC.

In addition to the aforementioned differences in contractile properties that we tested (e.g., EDL absolute force, soleus specific force, muscle morphology and fiber type composition), we identified a number of similarities. For example, the coefficient of variation (CV) in absolute force production of the EDL at the 100 Hz contraction was only 10.9% across strains. Similarly, the EDL specific force CV at 100 Hz was only 6.6%. Another notable similarity was fatigue resistance of both muscles, particularly in the early portions of the protocols. Importantly, our data indicate that strain may or may not impact measurements of skeletal muscle function. It is thus the responsibility of the investigator to test for strain-mediated differences in outcome measures of experimental designs that employ multiple mouse strains.

A limitation to present study is the utilization of only male mice as sexual dimorphisms in skeletal muscle physiology have been documented extensively by Dr. Dawn Lowe’s lab (Moran et al., 2005; Collins et al., 2019). Another limitation includes the absence of animal body mass measures. Differences in body masses across strains may have placed different loads on the EDL and/or soleus muscles, which would have affected individual muscle masses. Measures of muscle mass may have also been affected by removal of only the proximal and distal tendons as the weight of connective tissue located within the muscle midbelly may have varied across strains. Any variation in muscle mass would ultimately influence specific force calculations. A final limitation of note is the use of the same contractile protocols for the EDL and the soleus muscles. Although these were employed to standardize our measures, it is possible that subjecting the soleus muscles to 150 total contractions was insufficient to induce true fatigue. Future studies from our lab will include measurements of body mass and a greater number of contractions in the soleus fatigue protocol. We will also focus on a comparison of contractile properties in common female mouse strains.

Investigators working in fields such as obesity, diabetes, and cancer already recognize the importance of mouse strain as a variable that can influence experimental outcomes (Fisher-Wellman et al., 2016; Reilly, 2016; Li et al., 2020). There are examples in muscular dystrophy research where background strain was found to influence the developing pathology in muscle. Specifically, the commonly used mouse model of muscular dystrophy is on a BL10 background (C57BL10-mdx, common name = mdx) (van Putten et al., 2019). The mdx mouse exhibits repeated bouts of muscle degeneration and regeneration across its lifespan, but unexpectedly, the model does not mimic the severity of the human condition of Duchenne’s muscular dystrophy despite the same loss of the dystrophin protein (Fukada et al., 2010). However, if the same mutation in the dystrophin gene is moved to a different genetic strain, DBA/2J, the resulting muscle pathology better resembles the human condition (van Putten et al., 2019; Hammers et al., 2020). Similar results were seen when the gene encoding the sarcoglycan gamma subunit was deleted from the DBA/2J and also from the 129T2/SvEmsJ background (Heydemann et al., 2005) resulting in a more severe myopathy in the DBA/2J strain. The disparity in the pathology was traced to a genetic polymorphism in the TGF-β-binding protein 4 gene (Ltbp4) that occurs in the DBA/2J mice. In contrast, transferring the mutation in the dystrophin gene to the 129/Sv background lessens the development of muscle pathology (Calyjur et al., 2016). Collectively, the data point to the importance of knowing the background strain of the mice as unique genetic differences may synergize or antagonize an induced deletion or overexpression that drives the resulting phenotype. In addition, it also conceptually points to the importance of using the proper control mice to control for genetic differences, with the best option being the use of wild-type (WT) littermates as controls.

The availability of transgenic and knockout mouse models has increased dramatically over the last 10 years, which has coincided with an increased use of the Cre-LoxP system in mice. To deliver the Cre transgene in an *in vivo* fashion, mating of two different strains of mice is often required (Frontera and Ochala, 2015). Thus, it is possible that any detected alteration in physiological function of the muscle might not be the result of the gene activation or deletion induced by the flox recombination, but instead the mixing of two different strains (Frontera and Ochala, 2015). Consequently, careful consideration should be given to what constitutes the WT animal when developing mouse models that require cross-breeding. Best practices involve utilizing littermates that lack either the Cre transgene or the specific flox site as control mice, including appropriate lengths of backcrossing, and performing single nucleotide polymorphism (SNP) analysis to allow for genetic monitoring of the background strain. If the experimental design includes crossing of mice with different backgrounds, it is appropriate to employ SNP analysis to characterize the background strain of the mouse model developed by the investigators. Our lab has recently conducted SNP analysis to determine background strain on in two of our different transgenic strains, which allows us to inform interested groups of the exact genetic makeup of our models (Jackson et al., 2018). Finally, previous data from our lab suggest that purchasing animals from a vendor to act as WT controls could lead to inadvertent misinterpretation of collected results.

Collectively, our results demonstrate that the physiological phenotypes of the EDL and soleus muscles exhibit both similarities and differences across commonly used inbred mouse strains. The data highlight a broader concept of why consideration of mouse strain is important in experimental design and further emphasize the necessity of investigators to accurately depict their mouse strain during the publication process. For example, only describing the C57BL/6 does not provide a complete explanation since the mouse can exist in multiple different strains (i.e., C57BL/6J or C57BL/6NJ). For the muscle biology field to ensure rigor and reproducibility of future work, it is important to consider and define strain effects on the biological outcomes assessed in the experimental approach.

## Data Availability

The raw data supporting the conclusions of this article will be made available by the authors, without undue reservation.

## Supplementary material

Supplementary Figure 1
**(A–E)** Representative cross-sectional images of EDL muscles from various strains of mice. **(A)** BL6NJ, **(B)** BALB/cJ, **(C)** FVB/NJ, **(D)** BL6J, and **(E)** BL10. Sections were stained for dystrophin (yellow) and the following myosin heavy chain isoforms‐Type I (blue), Type IIa (green), Type IIx (black), and Type IIb (red).

Supplementary Figure 2
**(A–E)** Representative cross-sectional images of soleus muscles from various strains of mice. **(A)** BL6NJ, **(B)** BALB/cJ, **(C)** FVB/NJ, **(D)** BL6J, and **(E)** BL10. Sections were stained for dystrophin (yellow) and the following myosin heavy chain isoforms‐Type I (blue), Type IIa (green), Type IIx (black), and Type IIb (red).

Supplementary Figure 3
**(A–E)** Individual frequency distribution of all fibers measured in the EDL muscles from each strain. **(A)** BL6NJ, **(B)** BALB/cJ, **(C)** FVB/NJ, **(D)** BL6J, and **(E)** BL10.

Supplementary Figure 4
**(A–E)** Individual frequency distribution of all fibers measured in the soleus muscles from each strain. **(A)** BL6NJ, **(B)** BALB/cJ, **(C)** FVB/NJ, **(D)** BL6J, and **(E)** BL10.
