# Supplementary figures and images for: Isometric skeletal muscle contractile properties in common strains of male laboratory mice
Source: Front Physiol. 2022 Oct 4;13:937132. doi: 10.3389/fphys.2022.937132 (PMC9576934; doi:10.3389/fphys.2022.937132)

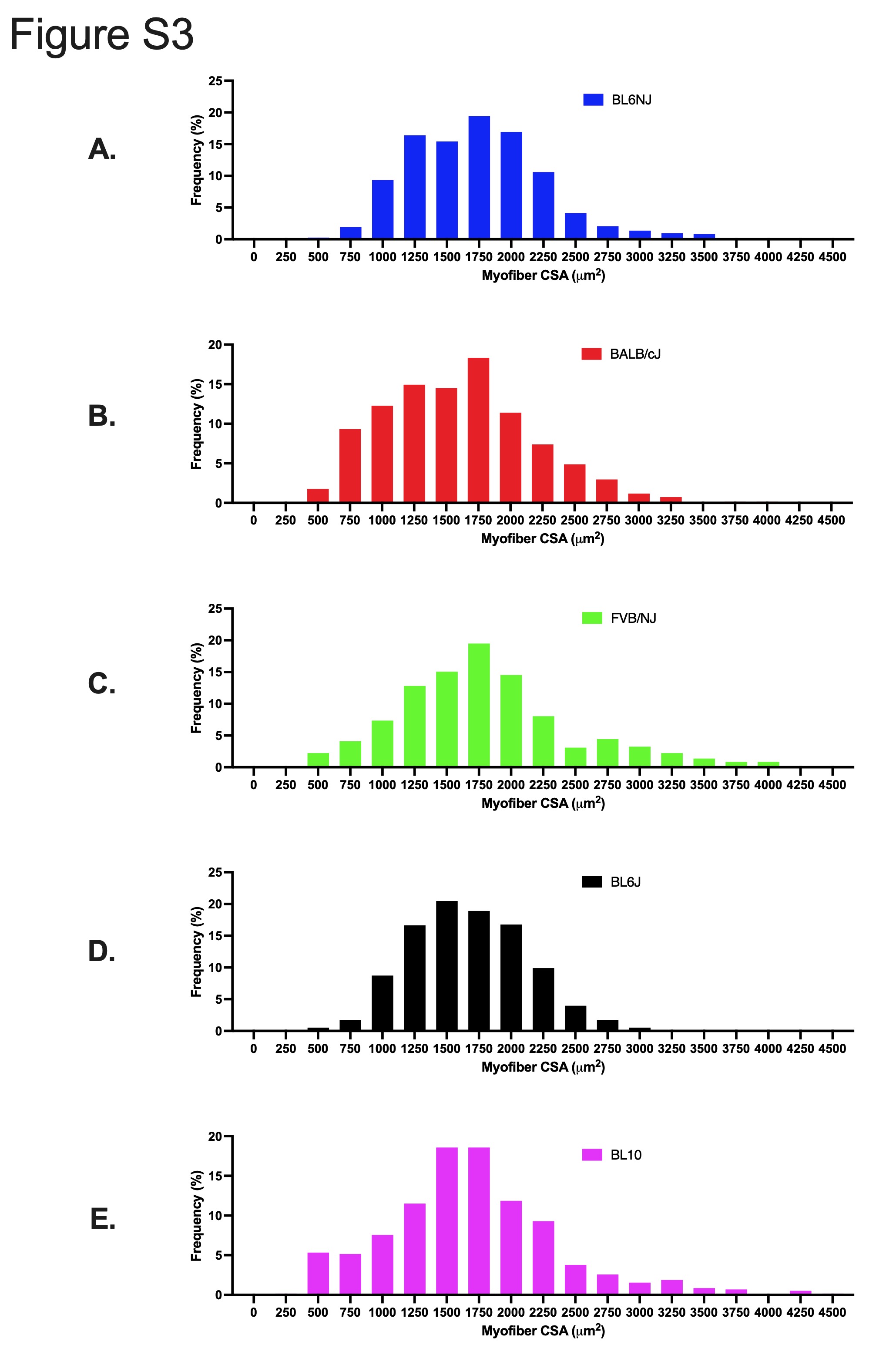

Supplement: Supplementary file 1 [file Image3.JPEG]

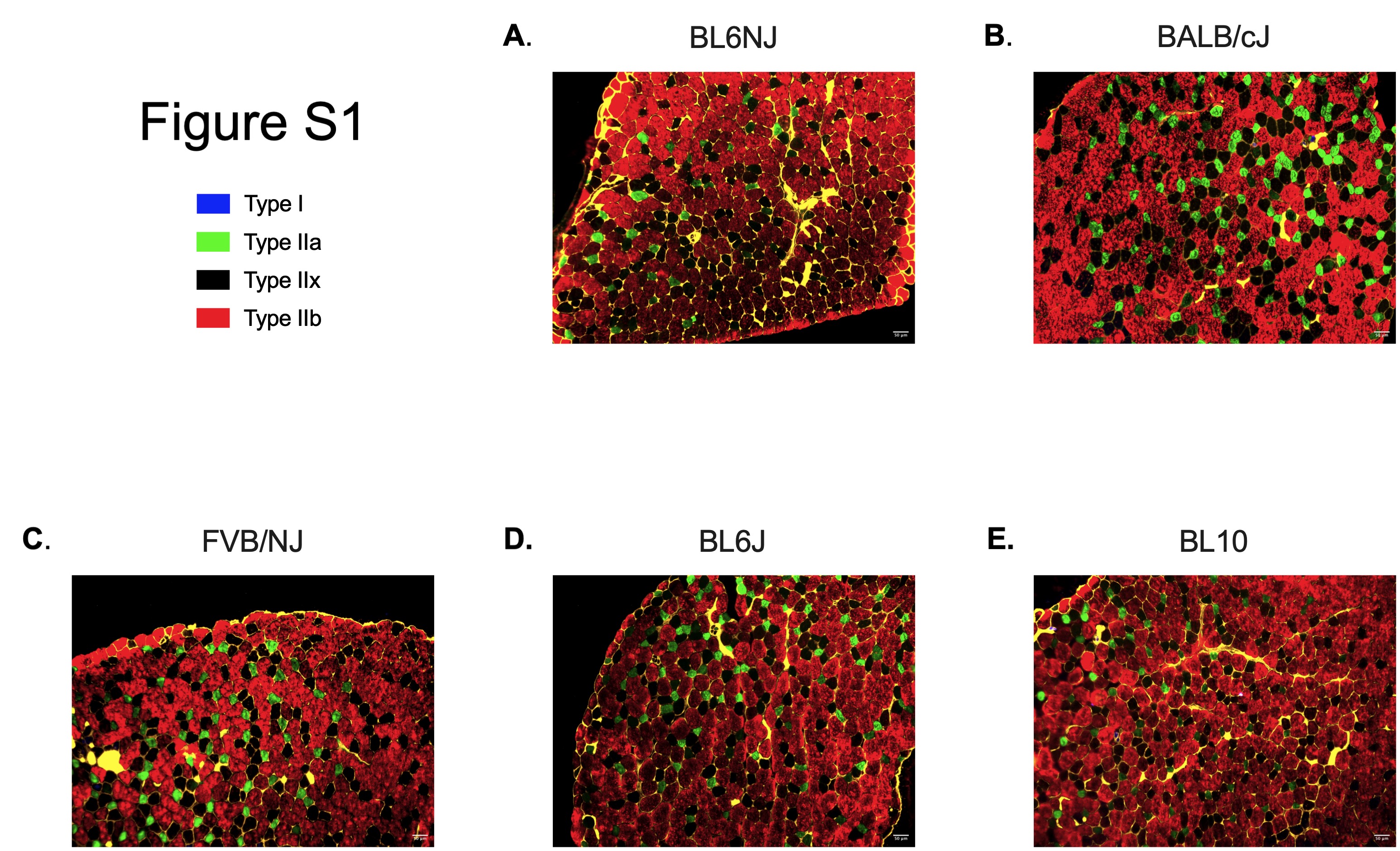

Supplement: Supplementary file 2 [file Image1.JPEG]

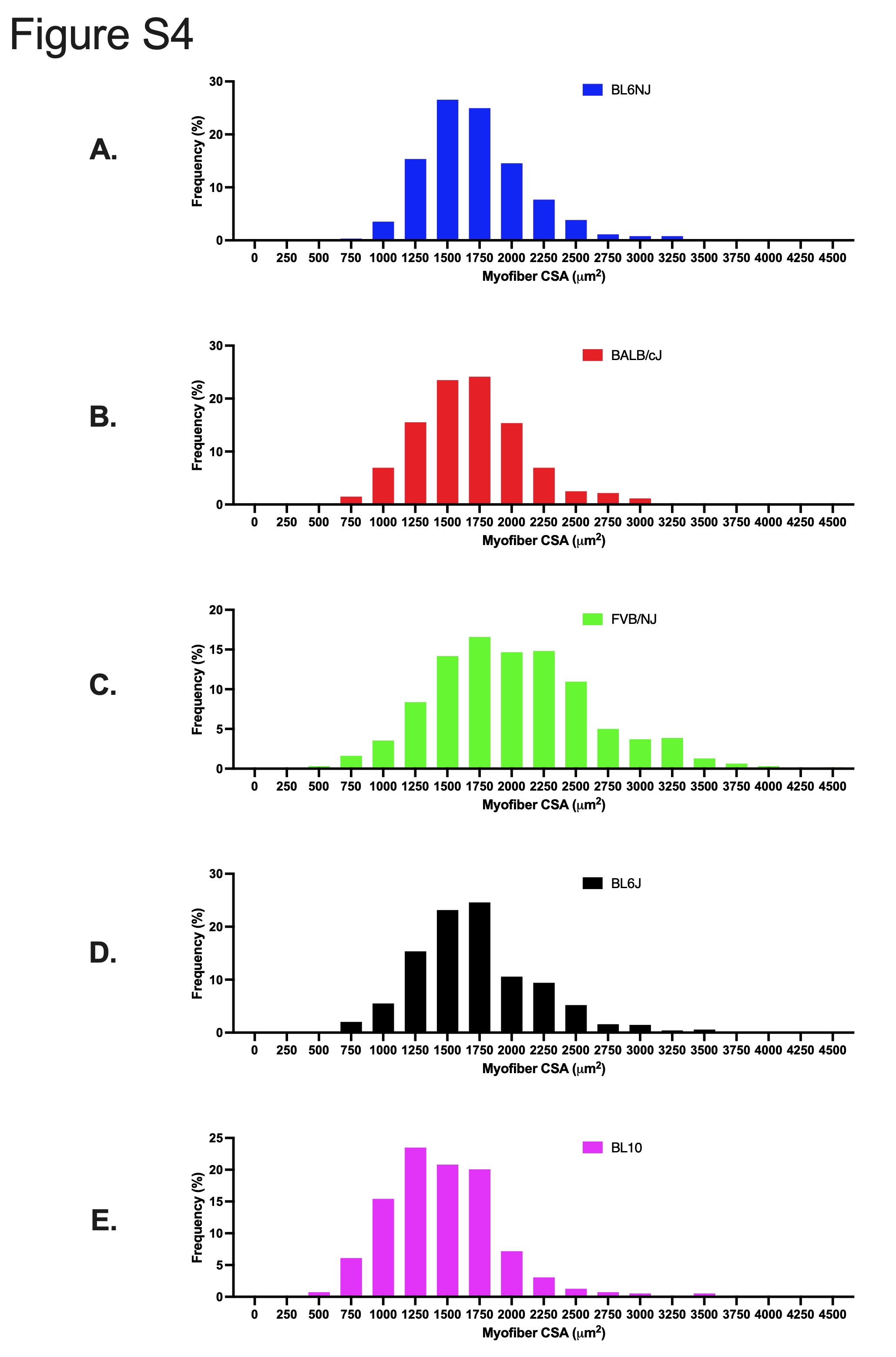

Supplement: Supplementary file 3 [file Image4.JPEG]

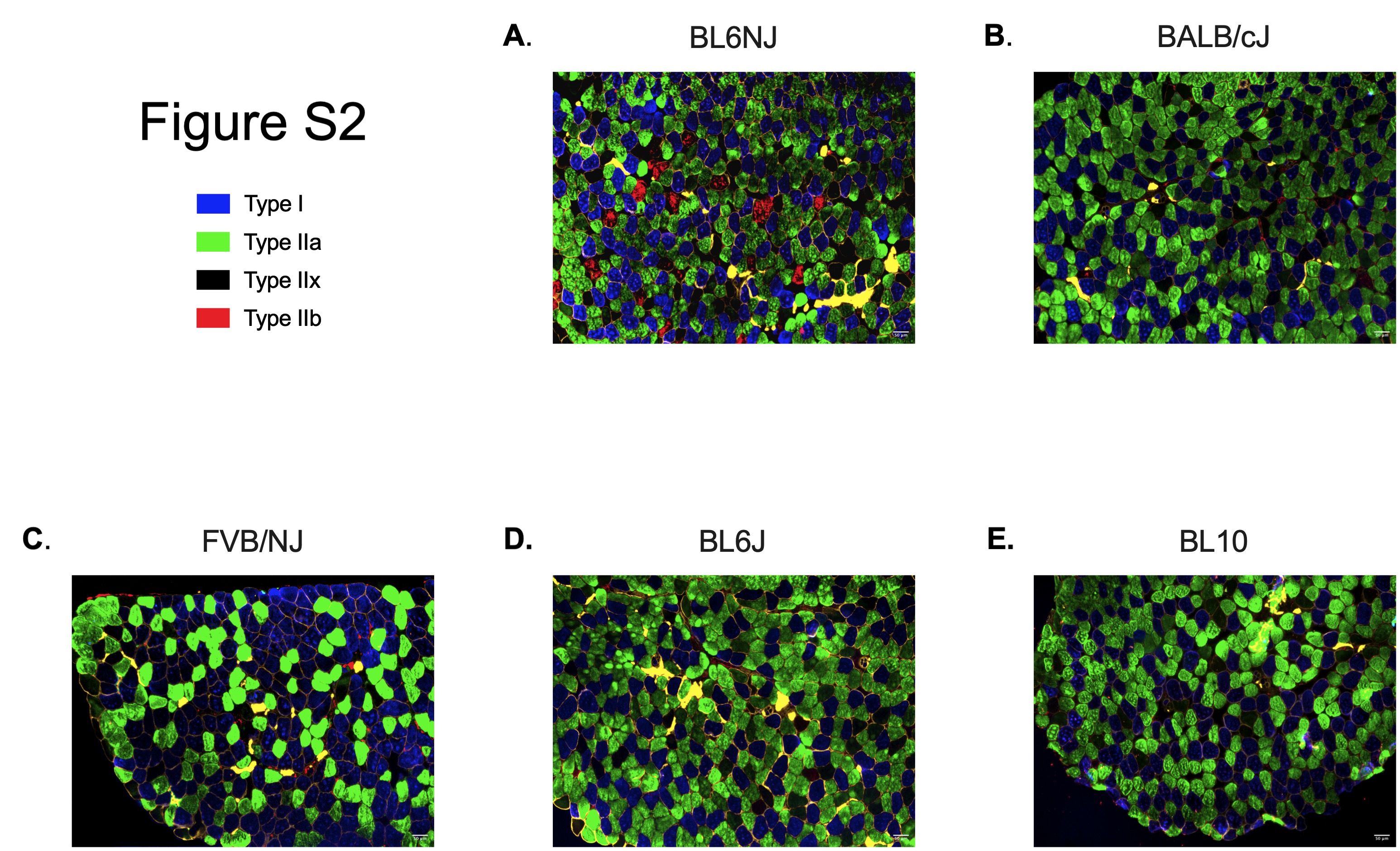

Supplement: Supplementary file 4 [file Image2.JPEG]
